# Four new *Ophiostoma* species associated with conifer- and hardwood-infesting bark and ambrosia beetles from the Czech Republic and Poland

**DOI:** 10.1007/s10482-019-01277-5

**Published:** 2019-05-28

**Authors:** Robert Jankowiak, Piotr Bilański, Beata Strzałka, Riikka Linnakoski, Agnieszka Bosak, Georg Hausner

**Affiliations:** 1 Department of Forest Pathology, Mycology and Tree Physiology, Institute of Forest Ecosystem Protection, University of Agriculture in Krakow, Al. 29 Listopada 46, 31-425 Kraków, Poland; 2 Department of Forest Protection, Entomology and Forest Climatology, Institute of Forest Ecosystem Protection, University of Agriculture in Krakow, Al. 29 Listopada 46, 31-425 Kraków, Poland; 3 Natural Resources Institute Finland (Luke), Latokartanonkaari 9, 00790 Helsinki, Finland; 4 Department of Microbiology, Buller Building 213, University of Manitoba, Winnipeg, R3T 2N2 Canada

**Keywords:** Conifers, Hardwoods, 4 New taxa, *Ophiostoma piceae* species complex

## Abstract

Fungi under the order Ophiostomatales (Ascomycota) are known to associate with various species of bark beetles (Coleoptera: Curculionidae: Scolytinae). In addition this group of fungi contains many taxa that can impart blue-stain on sapwood and some are important tree pathogens. A recent survey that focussed on the diversity of the Ophiostomatales in the forest ecosystems of the Czech Republic and Poland uncovered four putative new species. Phylogenetic analyses of four gene regions (ITS1-5.8S-ITS2 region, ß-tubulin, calmodulin, and translation elongation factor 1-α) indicated that these four species are members of the genus *Ophiostoma*. All four newly described species can be distinguished from each other and from closely related species based on DNA sequence comparisons, morphological characters, growth rates, and their insect associations. Based on this study four new taxa can be circumscribed and the following names are provided: *Ophiostoma pityokteinis* sp. nov., *Ophiostoma rufum* sp. nov., *Ophiostoma solheimii* sp. nov., and *Ophiostoma taphrorychi* sp. nov. *O. rufum* sp. nov. is a member of the *Ophiostoma piceae* species complex, while *O. pityokteinis* sp. nov. resides in a discrete lineage within *Ophiostoma s. stricto*. *O. taphrorychi* sp. nov. together with *O. distortum* formed a well-supported clade in *Ophiostoma s. stricto* close to *O. pityokteinis* sp. nov. *O. solheimii* sp. nov. groups within a currently undefined lineage A, which also includes *Ophiostoma grandicarpum* and *Ophiostoma microsporum*. This study highlights the need for more intensive surveys that should include additional countries of Central Europe, insect vectors and host tree species in order to elucidate *Ophiostoma* species diversity in this region.

**Electronic supplementary material:**

The online version of this article (10.1007/s10482-019-01277-5) contains supplementary material, which is available to authorized users.

## Introduction

The order Ophiostomatales includes seven well supported lineages represented by the following genera: *Aureovirgo*, *Ceratocystiopsis*, *Fragosphaeria*, *Graphilbum, Hawksworthiomyces*, *Raffaelea s. stricto*, and *Sporothrix*. Two additional major groups, for which monophyly is not well supported, are *Leptographium s. lato* and *Ophiostoma s. lato* (De Beer and Wingfield 2013; De Beer et al. 2016). The Ophiostomatales also contain some smaller lineages with uncertain taxonomic positions, such as lineages A, B, C and D (De Beer et al. 2016).

Species of *Ophiostoma* Syd. & P. Syd. (Sydow and Sydow 1919) reside in *Ophiostoma s. stricto* (Ophiostomatales, Ascomycota) (De Beer et al. 2016). Currently, *Ophiostoma s. stricto* includes six species complexes: *O. ulmi*, *O. pluriannulatum*, *O. ips*, *O. clavatum*, *O. minus*, and *O. piceae* species complexes (De Beer and Wingfield 2013; Linnakoski et al. 2016; Yin et al. 2016; Chang et al. 2019). The genus *Ophiostoma* currently includes nearly 40 described taxa, most of which are associated with phloem and wood-dwelling beetles. The most important morphological features that can be used to describe these fungi are ascomata with short to long necks, crescent to allantoid shaped ascospores, and pesotum-, hyalorhinocladiella- or sporothrix-like asexual morphs (De Beer and Wingfield 2013). Most *Ophiostoma* species produce spores in sticky droplets that can easily attach to the exoskeletons of their insect vectors (Malloch and Blackwell 1993). The genus *Ophiostoma* includes plant-associated species with varying degrees of pathogenicity. Most members are considered as non-pathogenic, especially in their endemic range, where they have co-evolved with their host tree species, and are mainly responsible for causing blue-stain in freshly exposed sapwood (Wingfield et al. 2017). However, some *Ophiostoma* species are highly virulent tree pathogens that have been responsible for tree death in natural as well as managed forest ecosystems (Harrington 1993). In many cases, pathogenicity and tree damage caused by these fungi are linked to their introduction into new areas (Loo 2009; Wingfield et al. 2015).

Members of the Ophiostomatales, that exist in symbiosis with bark beetles in Central Europe, have been mainly described from Austria (e.g. Kirisits 2001), Germany (e.g. Kirschner 2001), Poland (e.g. Siemaszko 1939; Jankowiak 2005, 2006, 2008; Jankowiak and Bilański 2013) and from a limited number of reports from the Czech Republic and Slovakia (e.g. Kotýnková-Sychrová 1966). These studies reported numerous species belonging to the Ophiostomatales that were in association with conifer- and hardwood-infesting bark beetles. However, the diversity of ophiostomatoid fungi associated with *Abies alba*, *Larix decidua* and hardwood trees are not well studied. For this reason, several comprehensive studies have been undertaken in recent years to explore the diversity of ophiostomatoid fungi in Central Europe (Jankowiak et al. 2017a, 2019). As part of a fungal diversity survey conducted in the Czech Republic and Poland (Jankowiak et al. 2017a, 2019) a total of 30 undescribed Ophiostomatales taxa associated with hardwood- and conifer-infesting beetles were uncovered. Until now, only six of these have been formally described as new species (Jankowiak et al. 2017b, 2018; Aas et al. 2018).

In this study, both morphological characters and DNA sequence data from the ITS region (ITS1-5.8S-ITS2), and three protein coding genes (β-tubulin, calmodulin, translation elongation factor 1-α) were analysed to a) characterise the four new species within *Ophiostoma s. lato*, and compare them to closely related known species within the Ophiostomatales, and b) to provide a formal description for these new species.

## Materials and methods

### Isolates and herbarium specimens

**Fig. 1:**
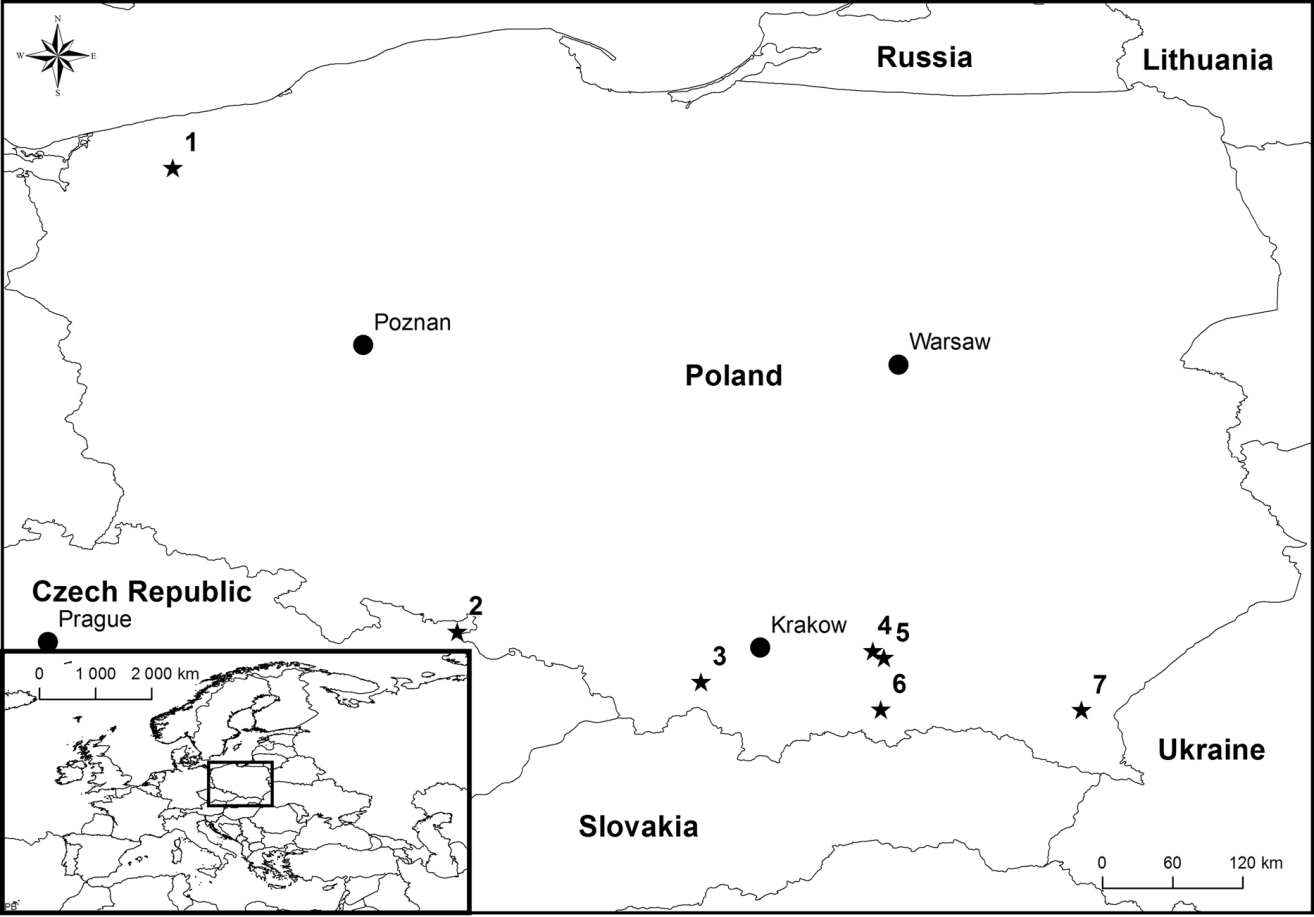
Geographic origins of isolates used in this study: 1—Resko, Poland (53°45′56.18″N, 15°25′19.25″E); 2—Albrechtice, Czech Republic (50°11′18.00″N, 17°36′39.90″E); 3—Mucharz, Poland (49°48′7.98″N, 19°29′19.50″E); 4—Wierzchosławice, Poland (50°2′21.06″N, 20°48′49.32″E); 5—Zbylitowska Góra, Poland (49°59′9.28″N, 20°53′55.27″E);. 6—Nawojowa, Poland (49°35′19.38″N, 20°52′28.49″E); 7—Rozpucie, Poland (49°34′59.92″N, 22°25′18.28″E). Dark grey—forests, light grey—sea

Bark beetles and galleries were collected during a study conducted by Jankowiak et al. (2017a, 2019). Fungal isolations were made from beetles collected in Poland, and this included the following beetle species: *Pityokteines vorontzowi*, *Pityokteines curvidens, Anisandrus dispar, Taphrorychus bicolor* and *Scolytus intricatus* (Fig. 1). In addition, materials investigated in this study included fungi that were isolated from *Ips cembrae* in the Czech Republic (Table 1). Fungal isolation strategy and the origin of some of the isolates used in this study have been described previously (Jankowiak et al. 2017a, 2019).

**Table 1:** Isolates used in the present study

Species	Isolate no.^a^				Host	Insect	Origin	GenBank accession no.			
	CMW	CBS	Herbarium	Other				ITS	βT	TEF1-α	CAL
Taxon1	52062^T^	144871^bT^	TUR207541^c^ (http://mus.utu.fi/TFU.207541)	KFL79LMD	*Larix decidua*	*Ips cembrae*	Albrechtice, CZ	MH837040	KY568440	KY568647	MH837063
*O. rufum* sp. nov.	52065	144872^b^	TUR207542 (http://mus.utu.fi/TFU.207542)	KFL67LMD	*L. decidua*	*I. cembrae*	Albrechtice, CZ	KY568153	KY568441	KY568648	MH837064
	52064	144873	TUR207543 (http://mus.utu.fi/TFU.207543)	KFL63bLMD	*L. decidua*	*I. cembrae*	Albrechtice, CZ	KY568154	KY568442	KY568649	MH837065
Taxon 2	52059	144874	TUR207544 (http://mus.utu.fi/TFU.207544)	KFL9209FJD	*Abies alba*	*Pityokteines vorontzowi*	Mucharz, PL	KY568155	KY568444	KY568650	MH837066
*O. pityokteinis* sp. nov.	52055	–		KFL8MFJD	*A. alba*	*Ptyokteines curvidens*	Nawojowa, PL	MH837041	KY568445	MH837053	MH837067
	–	–		KFL67KFJD	*A. alba*	*P. curvidens*	Rozpucie, PL	KY568156	KY568446	KY568651	MH837068
	52060	144875		KFL56KFJD	*A. alba*	*P. curvidens*	Rozpucie, PL	MH837042	KY568447	KY568652	MH837069
	52057	144876^b^	TUR207545 (http://mus.utu.fi/TFU.207545)	KFL4MFJD	*A. alba*	*P. curvidens*	Nawojowa, PL	MH837043	KY568448	KY568653	MH837070
	52058	144877		KFL35MFJD	*A. alba*	*P. curvidens*	Nawojowa, PL	MH837044	KY568449	KY568654	MH837071
	52054	144878		KFL2MbFJD	*A. alba*	*P. curvidens*	Nawojowa, PL	MH837045	KY568450	MH837054	MH837072
	52056^T^	144879^bT^	TUR207546^c^ (http://mus.utu.fi/TFU.207546)	KFL23MFJD	*A. alba*	*P. curvidens*	Nawojowa, PL	MH837046	KY568451	MH837055	MH837073
	52061	144880	TUR207547 (http://mus.utu.fi/TFU.207547)	KFL18214aFJD	*A. alba*	*P. curvidens*	Rozpucie, PL	KY568157	KY568452	KY568655	MH837074
Taxon 3	–	–		KFL7716RJTB	*Fagus sylvatica*	*Taphrorychus bicolor*	Rozpucie, PL	–	MH283332	MH283482	MH837076
*O. taphrorychi* sp. nov.	52042	144884	TUR207550 (http://mus.utu.fi/TFU.207550)	KFL7216RJTB	*F. sylvatica*	*T. bicolor*	Rozpucie, PL	–	MH283333	MH283483	–
	–	–		KFL7816RJTB	*F. sylvatica*	*T. bicolor*	Rozpucie, PL	–	MH283334	MH283484	–
	52043	144885	TUR 207551(http://mus.utu.fi/TFU.207551)	KFL5916RJTB	*F. sylvatica*	*T. bicolor*	Rozpucie, PL	MH837047	MH283335	MH837056	MH837077
	52044	144886		KFL6615RJTB	*F. sylvatica*	*T. bicolor*	Zbylitowska Góra, PL	MH837048	MH283336	MH837057	MH837078
	52049	144887	TUR207552 (http://mus.utu.fi/TFU.207552)	KFL36916RJTB	*F. sylvatica*	*T. bicolor*	Rozpucie, PL	MH283129	MH283337	MH283485	MH837079
	52046	144888	TUR207553 (http://mus.utu.fi/TFU.207553)	KFL37016RJTB	*F. sylvatica*	*T. bicolor*	Rozpucie, PL	MH837049	MH283338	MH837058	MH837080
	52047	144889^b^	TUR207554 (http://mus.utu.fi/TFU.207554)	KFL37116RJTB	*F. sylvatica*	*T. bicolor*	Rozpucie, PL	MH837050	MH283339	MH837059	MH837081
	52048	144890		KFL37816RJTB	*F. sylvatica*	*T. bicolor*	Rozpucie, PL	MH837051	MH283340	MH837060	MH837082
	–	–		KFL82316RJTB	*F. sylvatica*	*T. bicolor*	Rozpucie, PL	MH283130	MH283341	MH283486	MH837083
	–	–		KFL20614RJTB	*F. sylvatica*	*T. bicolor*	Rozpucie, PL	MH283131	MH283342	MH837061	–
	52045^T^	144891^bT^	TUR207555^c^ (http://mus.utu.fi/TFU.207555)	KFL20814aRJTB	*F. sylvatica*	*T. bicolor*	Rozpucie, PL	MH837052	MH283343	MH837062	MH837084
	–	–		KFL6415RJTB	*F. sylvatica*	*T. bicolor*	Zbylitowska Góra, PL	–	MH283344	MH283487	MH837085
Taxon 4	52050^T^	144881^bT^	TUR207548^c^ (http://mus.utu.fi/TFU.207548)	KFL1035c16RJAD	*Quercus robur*	*Anisandrus dispar*	Resko, PL	MH283134	MH283345	MH283488	MH837075
*O. solheimii* sp. nov.	52051	144882^b^	TUR207549 (http://mus.utu.fi/TFU.207549)	KFL104916RJAD	*Q. robur*	*A. dispar*	Resko, PL	MH283133	MH283346	MH283489	–
	52052	144883		KFL104316bRJAD	*Q. robur*	*A. dispar*	Resko, PL	MH283132	MH283347	MH283490	–
*O. grandicarpum*	52053	144892		KFL47616aRJSI	*Q. robur*	*Scolytus intricatus*	Wierzchosławice, PL	MH283116	MH283306	MH283470	MH837086

^a^CMW Culture Collection of the Forestry and Agricultural Biotechnology Institute (FABI), University of Pretoria, Pretoria, South Africa; CBS Westerdijk Fungal Biodiversity Institute, Utrecht, The Netherlands; TFU the TUR Herbarium of the University of Turku, Finland; KFL Culture collection of the Department of Forest Pathology, Mycology and Tree Physiology; University of Agriculture in Krakow, Poland; sequences generated from this study: MH837040–MH837086

^b^Isolates used in growth and morphological studies; ^c^holotype; ^T^Ex-type

All fungal isolates used in this study are listed in Table 1. The isolates are maintained in the culture collection of the Department of Forest Pathology, Mycology and Tree Physiology; University of Agriculture in Krakow, Poland. The ex-type isolates of the new species described in this study were deposited in the Westerdijk Fungal Biodiversity Institute (CBS), Utrecht, the Netherlands, and in the culture collection (CMW) of the Forestry and Agricultural Biotechnology Institute (FABI), University of Pretoria, South Africa. Herbarium specimens have been deposited in the Herbarium of the University of Turku (TUR), Finland. Taxonomic descriptions and nomenclatural data have been registered in MycoBank (www.MycoBank.org) (Robert et al. 2013).

### DNA extraction, PCR and sequencing

The fungal isolates were grown on 2% malt extract agar [MEA: 20 g Bacto™ malt extract (Becton–Dickinson and Company, Franklin Lakes, USA), 20 g agar (Bacto™ agar powder from Becton–Dickinson and Company, Franklin Lakes, USA), 1 l deionized water] in 90 mm plastic Petri dishes for 1–2 weeks prior to DNA extraction. DNA was extracted using the Genomic Mini AX Plant Kit (A&A Biotechnology, Gdynia, Poland) according to the manufacturer’s protocol.

Four loci, including ITS1–5.8 S–ITS2 (ITS), beta-tubulin (βT), calmodulin (CAL) and translation elongation factor 1-alpha (TEF1-α) were amplified for sequencing and phylogenetic analyses. Primers used in this study were as follows: ITS 1-F (Gardes and Bruns 1993) and ITS4 (White et al. 1990) for the ITS region, Bt2a (Glass and Donaldson 1995) or T10 (O’Donnell and Cigelnik 1997) and Bt2b (Glass and Donaldson 1995) for βT, CL2F and CL2R (Duong et al. 2012) for CAL, and F-728F (Carbone and Kohn 1999) and EF2 (O’Donnell et al. 1998) for TEF1-α.

DNA fragments were amplified in a 25 µL reaction mixture containing 0.25 µL of Phusion High-Fidelity DNA polymerase (Finnzymes, Espoo, Finland), 5 µL Phusion HF buffer (5x), 0.5 µL of dNTPs (10 mM), 0.75 µL DMSO (100%) and 0.5 µL of each primer (25 µM). Amplification reactions were performed in the LabCycler Gradient thermocycler (Sensoquest Biomedical Electronics GmbH, Germany). Amplification of the various loci was performed under the following conditions: a denaturation step at 98 °C for 30 s was followed by 35 cycles of 5 s at 98 °C, 10 s at 52–64 °C (depending on the primer melting temperature and fungal species) and 30 s at 72 °C, and a final elongation step at 72 °C for 8 min. The PCR products were visualized under UV light on a 2% agarose gel stained with Midori Green DNA Stain (Nippon Genetic Europe).

Amplified products were sequenced with the BigDye^®^ Terminator v 3.1 Cycle Sequencing Kit (Applied Biosystems, Foster City, CA, USA) and the products were resolved with a ABI PRISM 3100 Genetic Analyzer (Applied Biosystems), at the DNA Research Centre (Poznań, Poland) using the same primers that were used for the PCR. The sequences (Table 1) were compared with sequences retrieved from GenBank using the BLASTn algorithm (Altschul et al. 1990). Newly obtained sequences were deposited in NCBI GenBank (Table 1).

### Phylogenetic analyses

**Fig. 2:**
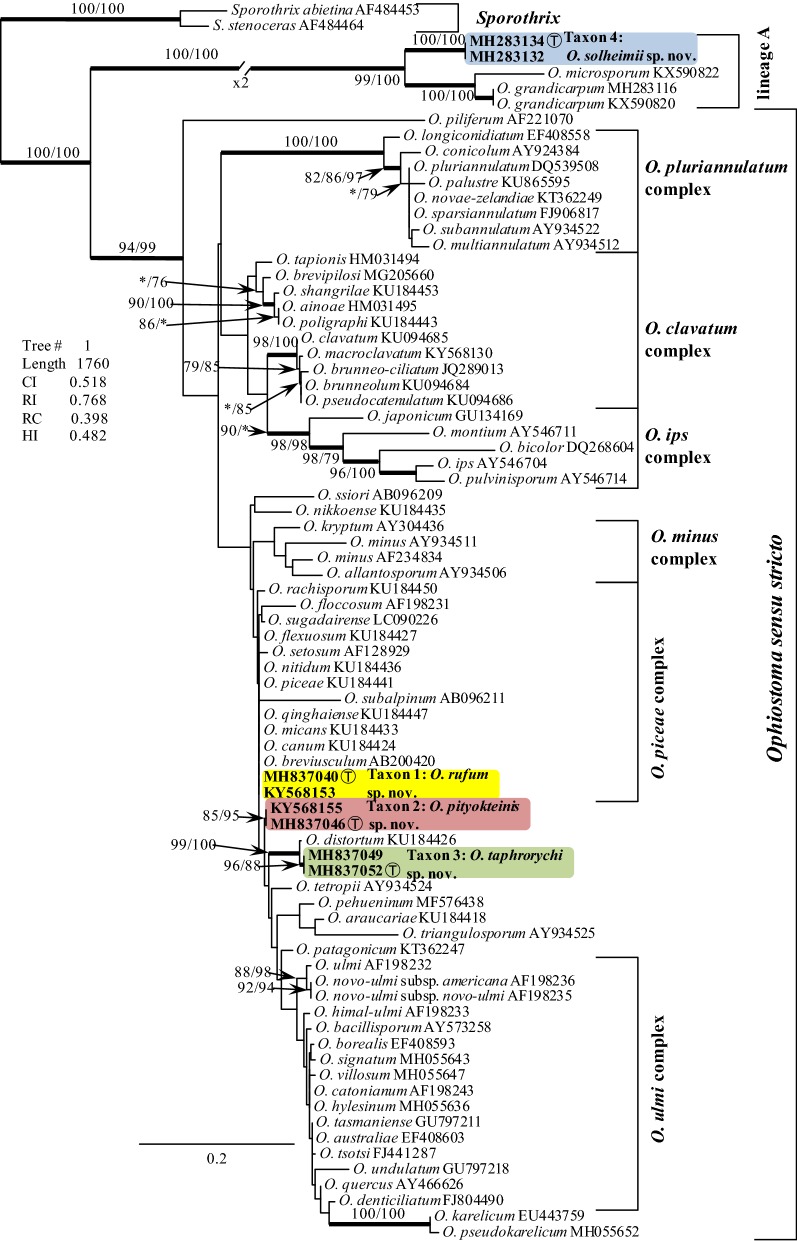
Tree topology based on ML analysis for species of *Ophiostoma s. lato* generated from the ITS DNA sequence data. Bootstrap values shown at nodes ≥ 75% for ML and Maximum Parsimony (MP) analyses are presented as follows: ML/MP. Bold branches indicate posterior probabilities values ≥ 0.95 were obtained from Bayesian Inference (BI) analyses. The symbol * indicates bootstrap values < 75%. The tree is drawn to scale (see bar) with branch length measured in the number of substitutions per site. Taxon 1—*Ophiostoma rufum* sp. nov., Taxon 2—*Ophiostoma pityokteinis* sp. nov., Taxon 3—*Ophiostoma taphrorychi* sp. nov., Taxon 4—*Ophiostoma solheimii* sp. nov. T—Ex-type

**Fig. 3:**
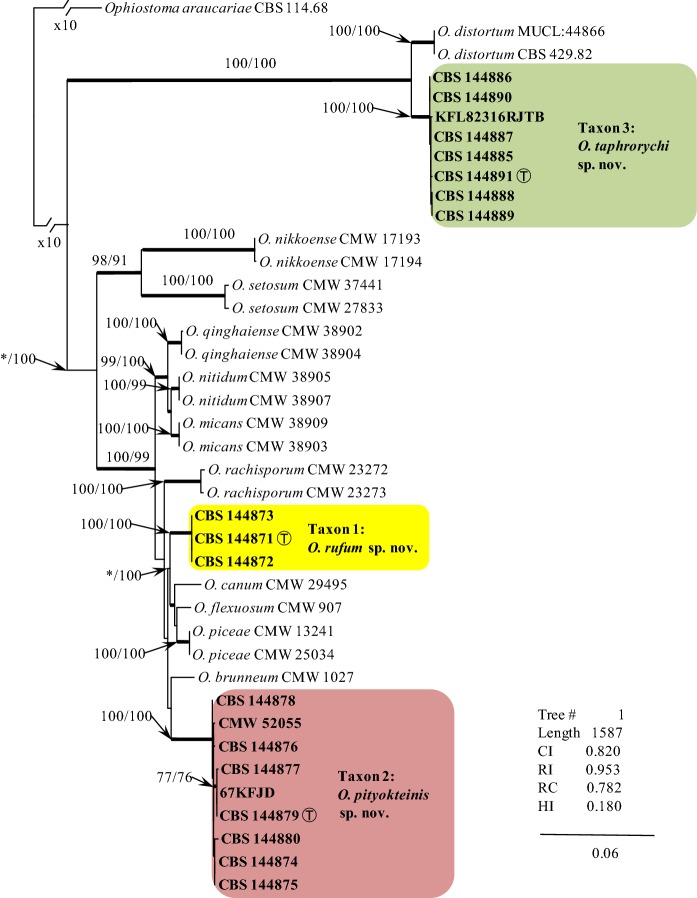
ML based tree topology for species in the *Ophiostoma piceae* species complex generated from the DNA sequences of the combined (concatenated) dataset including the ITS region and three protein coding gene sequences (βT, CAL, TEF1-α**)**. Bootstrap values ≥ 75% for ML and Maximum Parsimony (MP) analyses are presented at nodes as follows: ML/MP. Bold branches indicate posterior probabilities values ≥ 0.95 as obtained from Bayesian Inference (BI) analyses. The symbol * indicates bootstrap values < 75%. The tree is drawn to scale (see bar) with branch length measured in the number of substitutions per site. Taxon 1—*Ophiostoma rufum* sp. nov., Taxon 2—*Ophiostoma pityokteinis* sp. nov., Taxon 3—*Ophiostoma taphrorychi* sp. nov. T—Ex-type

BLAST searches using the BLASTn algorithm were performed to retrieve similar sequences from GenBank (http://www.ncbi.nlm.nih.gov) and accession numbers for these sequences are presented in the corresponding phylogenetic trees (Figs. 2 and 3, S1-S4). Datasets were curated with the Molecular Evolutionary Genetic Analysis (MEGA) v6.06 program (Tamura et al. 2013). The ITS dataset included all available sequences for reference species in *Ophiostoma s. lato* that could be retrieved from GenBank (Fig. 2) to show the placement of our isolates within this genus. The outgroup taxon for the ITS dataset analysis was *Sporothrix abietina* and *S. stenoceras*. The three protein coding gene regions were sequenced for 29 (βT, and TEF1-α) and 25 (CAL) of our isolates (Table 1). Datasets were analysed individually and with regards to the protein coding sequences as concatenated constructs. Sequence alignments were performed using the online version of MAFFT v7 (Katoh and Standley 2013). The ITS, ßT, CAL and TEF1-α datasets were aligned using the E-INS-i strategy with a 200PAM/κ = 2 scoring matrix, a gap opening penalty of 1.53 and an offset value of 0.00. The alignments were checked manually with BioEdit v.2.7.5 (Hall 1999), and for the protein coding regions the alignments were compared with gene maps previously published by Aas et al. (2018) to ensure that introns and exons were aligned appropriately. The resulting alignments and trees were deposited into TreeBASE (TB2:S24036).

Phylogenetic trees were inferred for each of the datasets using three different methods: Maximum Likelihood (ML), Maximum Parsimony (MP) and Bayesian Inference (BI). For ML and BI analyses, the best-fit substitution models for each aligned dataset were established using the corrected Akaike Information Criterion (AICc) in jModelTest 2.1.10 (Guindon and Gascuel 2003; Darriba et al. 2012). ML analyses were carried out with PhyML 3.0 (Guindon et al. 2010), utilizing the Montpelier online server (http://www.atgc-montpellier.fr/phyml/). The ML analysis included bootstrap analysis (1000 bootstrap pseudoreplicates) in order to assess node support values and the overall reliability of the tree topology.

MP analyses were performed with PAUP* 4.0b10 (Swofford 2003). Gaps were treated as fifth state characters. Bootstrap analysis (1000 bootstrap replicates) was conducted to determine the levels of confidence for the nodes within the inferred tree topologies. Tree bisection and reconnection (TBR) was selected as the branch swapping option. The tree length (TL), Consistency Index (CI), Retention Index (RI), Homoplasy Index (HI) and Rescaled Consistency Index (RC) were recorded for each analysed dataset after the trees were generated.

BI analyses using Markov Chain Monte Carlo (MCMC) methods were carried out with MrBayes v3.1.2 (Ronquist and Huelsenbeck 2003). The four MCMC chains were run for 10 million generations applying the best-fit model. Trees were sampled every 100 generations, resulting in 100,000 trees. The Tracer v1.4.1 program (Rambaut and Drummond 2007) was utilized to determine the burn-in value for each dataset. The remaining trees were utilized to generate a 50% majority rule consensus tree, which allowed for calculating posterior probability values for the nodes.

### Morphology, growth studies and mating tests

Morphological characters were examined for selected isolates and for the herbarium specimens chosen to represent the type specimens for the newly proposed species. Cultures were grown on 2% MEA with or without host tree twigs to induce potential ascocarp formation. Autoclaved twigs with bark were positioned in the centre of the MEA agar plates. Fungal cultures were derived from single spores, and crossings were made following the technique described by Grobbelaar et al. (2010). To encourage the production of ascomata for species descriptions, single conidial isolates were crossed in all possible combinations. Cultures were incubated at 25 °C and monitored regularly for the appearance of fruiting structures.

Morphological features were examined by mounting materials in 80% lactic acid on glass slides, and observing various fruiting structures using a Nikon Eclipse 50*i* microscope (Nikon^®^ Corporation, Tokyo, Japan) with an Invenio 5S digital camera (DeltaPix^®^, Maalov, Denmark) to capture photographic images. Microscopy was done as previously described by Kamgan Nkuekam et al. (2011). Colour designations were based on the charts of Kornerup and Wanscher (1978).

For each taxonomically relevant structure fifty measurements were made, whenever possible, with the Coolview 1.6.0 software (Precoptic^®^, Warsaw, Poland). Averages, ranges and standard deviations were calculated for the measurements, and these are presented in the format ‘(min–)(mean − SD)–(mean + SD)(–max)’.

Growth characteristics for the four newly proposed species were determined by analysing the radial growth for four isolates in pure culture that represent each of the studied species (Table 1). Agar disks (5 mm diam.) were cut from actively growing margins of fungal colonies for each of the tested isolates and these disks were placed in the centre of plates containing 2% MEA. Four replicate plates for each of the isolates studied were incubated at 5, 10, 15, 20, 25, 30 and 35 °C. Colony diameters (two measurements per plate) were determined 7 d after inoculation and radial growth rates were calculated as mm/d.

## Results

### Morphological characteristics

The four new taxa showed differences with regards to growth rates in culture and colour differences ranging from rust brown, grey brown, to olive brown (Table 2). Taxon 1 and Taxon 2 produced abundant synnemata that were arranged either singly or in groups topped with cream-white mucilaginous spore drops. A sporothrix-like synanamorph was also present in cultures of Taxon 1. In addition, Taxon 3 and Taxon 4 produced hyalorhinocladiella-like asexual morphs. A sexual state was induced in Taxon 3 and 4. Sexual states were not observed for Taxon 1 and Taxon 2 in any of the crosses done between different isolates. Morphological differences among these new taxa are listed in Table 2, and discussed in the Notes under the new species descriptions in the Taxonomy section.

**Table 2:** Morphological comparisons of the novel taxa

Species	Taxon 1	Taxon 2	Taxon 3	Taxon 4
Sexual state	Unknown	Unknown	Present	Present
Ascomata base			(62–)88–130(–169)	(299–)329–457(–548)
Ascomatal neck length (μm)			(347–)444–559(–632)	(1192–)1627–2218(–2569)
Ascospore shape			Allantoid in side view, elliptical in front view	Orange-section
Ascospore size excluding sheath (in side view, μm)			(2.8–)3–3.6(–4) × (0.8–)1–1.3(–1.5)	(2.5–)2.6–3.1(–3.5) × (0.8–)1.1–1.4(–1.6)
Ascospore size excluding sheath (in side view, μm)			(2.8–)3.1–3.7(–4) × (1–)1.1–1.5(–1.7)	(2.3–)2.7–3.2(–3.8) × (0.8–)1.1–1.6(–1.9)
Asexual state	Pesotum-like, sporothrix-like	Pesotum-like	Hyalorhinocladiella-like	Hyalorhinocladiella-like
Synnematal length (μm)	(261–)506–1001(–1183)	(187–)265–511(–698)	–	–
Conidial shape	Oblong to curved	Obovoid	Oblong elliptical, obovoid to bacilliform, sometimes allantoid	Allantoid in side view; ellipsoidal in face view
Conidial size (μm)	(2.5–)3.3–4.5(–6.2) × (1.2–)1.4–1.7(–2.2)	(3.2–)3.5–4.1(–4.5) × (0.9–)1.2–1.6(–1.8)	(3.3–)4.2–5.4(–6.6) × (0.8–)0.9–1.3(–1.7)	allantoid: (2.9–)3.7–5.1(–6.3) × (0.8–)1.1–1.7(–2.2); ellipsoidal: (3.1–)3.9–4.8(–5.3) × (0.9–)1.2–1.8(–2.1)
Growth rate (mm d^−1^)	2.2 (± 0.1)	3.9 (± 0.1)	4.2 (± 0.1)	4.0 (± 0.1)
Optimal growth temp on MEA	25 °C	25 °C	20 °C	25 °C
Colony colour	Brownish orange to a rust brown	Coffee to dark brown	Brown	Olive brown
Habitat	Coniferous forest	Coniferous forest	Hardwood forest	Hardwood forest
Host	*L. decidua*	*A. alba*	*Fagus sylvatica*	*Quercus robur*
Insect	*Ips cembrae*	*Pityokteines curvidens*, *Pityokteines vorontzowi, Cryphalus piceae*	*Taphrorychus bicolor*	*Anisandrus dispar*
Distribution	Czech Republic	Poland	Poland	Poland

**Fig. 4:**
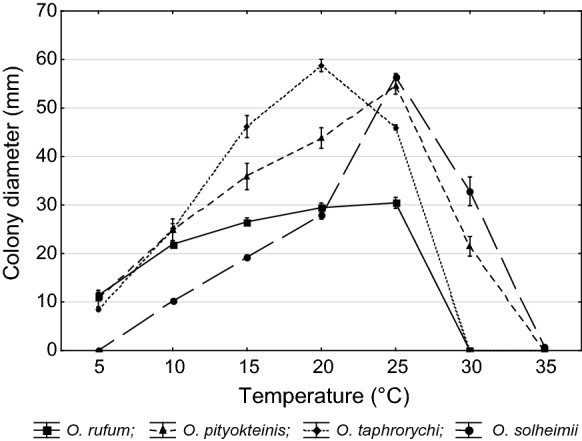
Comparison of mean growth on MEA (two isolates per tested species, ± standard deviation) of Taxa 1–4 held at different temperatures (grown 7 d in the dark)

The optimal growth temperature for Taxon 3 was at 20 °C, and at 25 °C for Taxon 1, 2 and 4. For all isolates the growth rate was minimal at 5 °C except for Taxon 4 which failed to grow at this temperature. No growth was observed at 30 °C for Taxon 1 and 3 and all Taxa failed to grow at 35 °C except for Taxon 4 (Fig. 4).

### Phylogenetic analyses

Alignments for the ITS, βT, CAL, TEF1-α sequences and the concatenated combined dataset contained 759, 400 (for βT *O. piceae* species complex), 503 (for βT lineage A), 936, 755 (for TEF1-α *O. piceae* species complex), 643 (for TEF1-α lineage A) and 2356 characters (including gaps), respectively. The exon/intron arrangement of the βT *O. piceae* species complex data included exons 3, 4, and 5/6, interrupted with introns 3 and 4, but lacking intron 5. The exon/intron arrangement of the βT lineage A data included exons 3, 4/5, and 6, interrupted with introns 2, 3 and 5, but lacking intron 4. The aligned TEF1-α gene region consisted of intron 3 and exons 4/5/6, while lacking introns 4 and 5. The alignment of the CAL dataset contained exons 2, 3, 4 and 5/6, interrupted with introns 2, 3, 4 and 6, while lacking intron 5.

The best evolutionary substitution model for ITS, βT, CAL, TEF1-α datasets was GTR + G. Except for the TEF1-α for lineage A dataset, for which the best model was GTR + I. The best evolutionary substitution model for the combined ITS, βT, CAL, TEF1-α datasets was GTR + I+G. The burn-in values in BI analyses for all data matrices were 25% of the trees.

The ITS tree shows the placement of the Czech and Polish isolates (referred to as Taxon 1 to Taxon 4) within the Ophiostomatales (Fig. 2). Taxa 1-3 resided among sequences representing species that are members of *Ophiostoma s. stricto*, while Taxon 4 is grouped with other species in the lineage A (Fig. 2) (De Beer et al. 2016). Taxa 1-3 appear to group closely with members of the *O. piceae* species complex. Taxon 1 grouped within the *O. piceae* species complex, while Taxa 2 and 3 formed two adjacent lineages peripheral to the *O. piceae* species complex (Fig. 2). The lineage that includes Taxon 3 includes the ex-type isolate of *O. distortum* (Fig. 2). Strains of Taxon 1 had ITS sequences that were identical with ITS sequences noted in most members of the *O. piceae* species complex. Taxon 4 grouped among members of the lineage A, which includes *O. grandicarpum* and *O. microsporum* (Fig. 2). This taxon had unique ITS sequences compared with *O. grandicarpum* and *O. microsporum*.

The BI, MP, ML phylogenetic analyses of the aligned protein-coding datasets (βT, CAL, TEF1-α and combined) for members of the *O. piceae* species complex yielded trees with different topologies (Fig. 3, Figs. S1–S3). In the βT, CAL and TEF1-α trees (Figs. S1-S3), Taxa 1-3 formed well-supported lineages that clearly separated these four newly proposed species from all the other known species in the *O. piceae* species complex and other closely related species. The only exception was Taxon 3, which had differences in the βT sequence compared to the *O. distortum* βT sequence, but the node lacked statistical support (Fig. S1). However, the combined analyses of the βT, CAL and TEF1-α datasets clearly distinguish Taxa 1-3 into separate lineages within *Ophiostoma s. stricto* (Fig. 3). Analyses of the βT, CAL and TEF1-α data grouped isolates of Taxon 4 in lineage A together with *O. macrosporum* and *O. grandicarpum* (βT) and *O. grandicarpum* (CAL and TEF1-α), corresponding to the ITS tree. This taxon formed a well-supported lineage that is clearly distinct from *O. macrosporum* and *O. grandicarpum* (Fig. S4).

### Taxonomy

The morphological characterization and phylogenetic comparisons based on four genetic loci, showed that four taxa associated with bark beetles from the Czech Republic and Poland (Taxa 1 to 4) are distinct from each other and from other known taxa in *Ophiostoma s. lato* and, therefore, are described here as new species. They are described as follows:

### Taxon 1

**Fig. 5:**
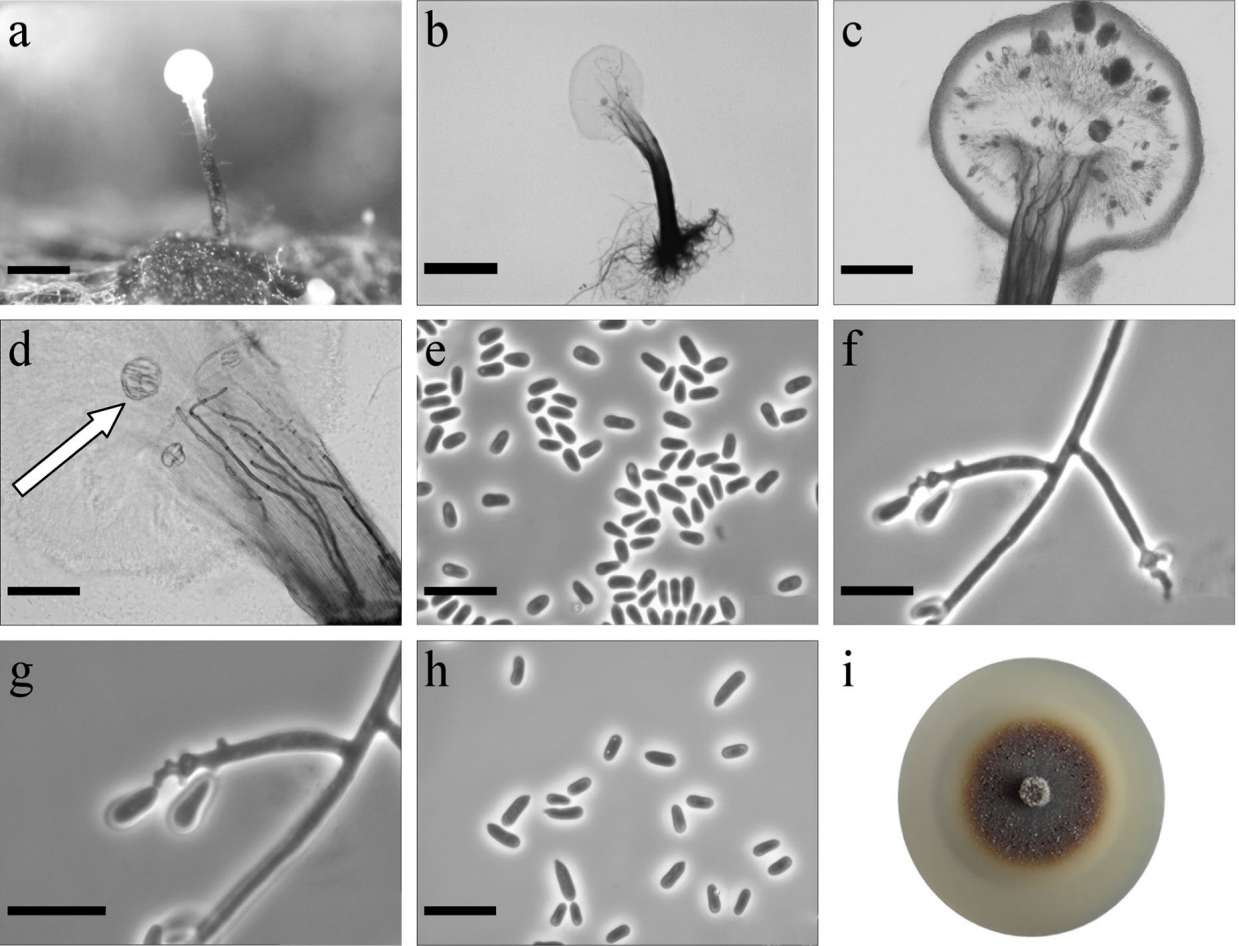
Morphological characters of *Ophiostoma rufum* sp. nov. (CBS 144871, Taxon 1). **a** Synnematous asexual state on wood tissue; **b** conidiophores; **c** conidiogeneous apparatus, arrow shows crystal-like structures; **d** conidiogeneous cells, arrow shows crystal-like structures; **e** conidia; **f**, **g** conidiophore of sporothrix-like asexual state with denticles of conidiogenous cell; **h** primary and secondary conidia; **i** fourteen-day-old culture on MEA. Scale bars: **a** = 250 μm, **b** = 250 μm, **c** = 100 μm, **d** = 50 μm, **e** = 10 μm, **f** = 10 μm, **g** = 10 μm, **h** = 10 μm

*Ophiostoma rufum* R. Jankowiak & P. Bilański, sp. nov. (Figure 5) MycoBank: MB830195.

*Etymology*: The epithet rufum, referring to the rust brown colony on MEA.

*Sexual state* not observed. *Asexual states*: pesotum-like and sporothrix-like. Pesotum-like: *Conidiophores* macronematous, synnematous, abundant in culture, synnemata occurring singly or in groups (261–)506–1001(–1183) μm long including conidiogenous apparatus (Fig. 5a, b). Stipe expanding towards both the apex and the base, dark brown at base, becoming paler toward apex (194–)422–881(–1018) μm long (26–)32–64(–116) μm wide at base, and (36–)37–117(–247) μm wide at apex. Crystal-like structures often occur in the upper part of the stipe (Fig. 5c d). *Conidiogenous cells* (8–)12.5–19.3(–24) × (1.4–)1.5–1.9(–2) μm (Fig. 5 d). *Conidia* hyaline, one-celled, smooth, oblong, sometimes slightly curved (2.5–)3.3–4.5(–6.2) × (1.2–)1.4–1.7(–2.2) μm aggregating into a cream-white mucilaginous spore drop (Fig. 5e). Synnematous asexual morphs frequently observed on different agar media with larch twigs. Asexual morph attached to substrate by brown rhizoid-like hyphae.

Sporothrix-like *Conidiophores* mononematous, micronematous, hyaline (4–)14.7–45.1(–80) μm long and (1.6–)2–2.6(–3) μm wide at the base, denticulate giving rise to primary conidia (Fig. 5f, g). Denticles terminated. *Primary conidia* non-septate, hyaline, clavate or fusiform (7.2–)8.9–12.4(–15.2) × (2–)2.5–3.1(–3.4) μm, sometimes producing denticles and giving rise to secondary conidia. *Secondary conidia* hyaline, smooth, one-celled, obovoid with a pointed base, sometimes slight curved (2–)4.5–6.7(–8) × (1.8–)1.9–2.5(–2.8) μm (Fig. 5h).

*Culture characteristics* Colonies with optimal growth at 25 °C on 2% MEA with radial growth rate 2.2 (± 0.1) mm/d, no growth occurred at 30 and 35 °C. Colonies brownish orange to a rust brown, with smooth margins (Fig. 5i). Reverse rust brown. Hyphae pale yellow to olive yellow in colour (Kornerup and Wanscher 1978), smooth, submerged in the medium and aerial mycelium abundant, not constricted at the septa, 0.6–5.3 (mean 1.8 ± 1.3) µm diam.

- Habitat: coniferous forest dominated by *Larix decidua*
- Host tree: *Larix decidua*
- Insect vectors: *Ips cembrae*
- Distribution: Czech Republic

*Type material* CZECH REPUBLIC, Albrechtice, in coniferous forest dominated by *L. decidua*, from *Ips cembrae* galleries established on *L. decidua*, collector *K. Lukášová*, 12 May 2014. Holotype dried specimen TUR 207541 (http://mus.utu.fi/TFU.207541), ex-holotype living culture CBS 144871 = CMW 52062.

*Notes* This species is most closely related to *O. breviusculum* (Chung et al. 2006) based on the phylogenetic analyses of the ITS, and βT sequences (Figs. 2, S1). However, the DNA sequences of βT and TEF1-α (Figs. S1, S2) are unique and clearly suggested that *O. rufum* is distinct from *O. breviusculum*, and other species of the *O. piceae* species complex.

Morphologically, *O. breviusculum* can be distinguished from this new species by having shorter synnemata. *Ophiostoma rufum* differs from *O. breviusculum* by the presence of crystal-like structures in the upper part of the stipe. In addition, *O. breviusculum* forms brown to dark brown colonies, while the new taxon has colonies displaying brownish orange to rust brown colours.

*Ophiostoma rufum* was infrequently isolated from *L. decidua* in association with *Ips cembrae* in Czech Republic (Jankowiak et al. 2017a).

### Taxon 2

**Fig. 6:**
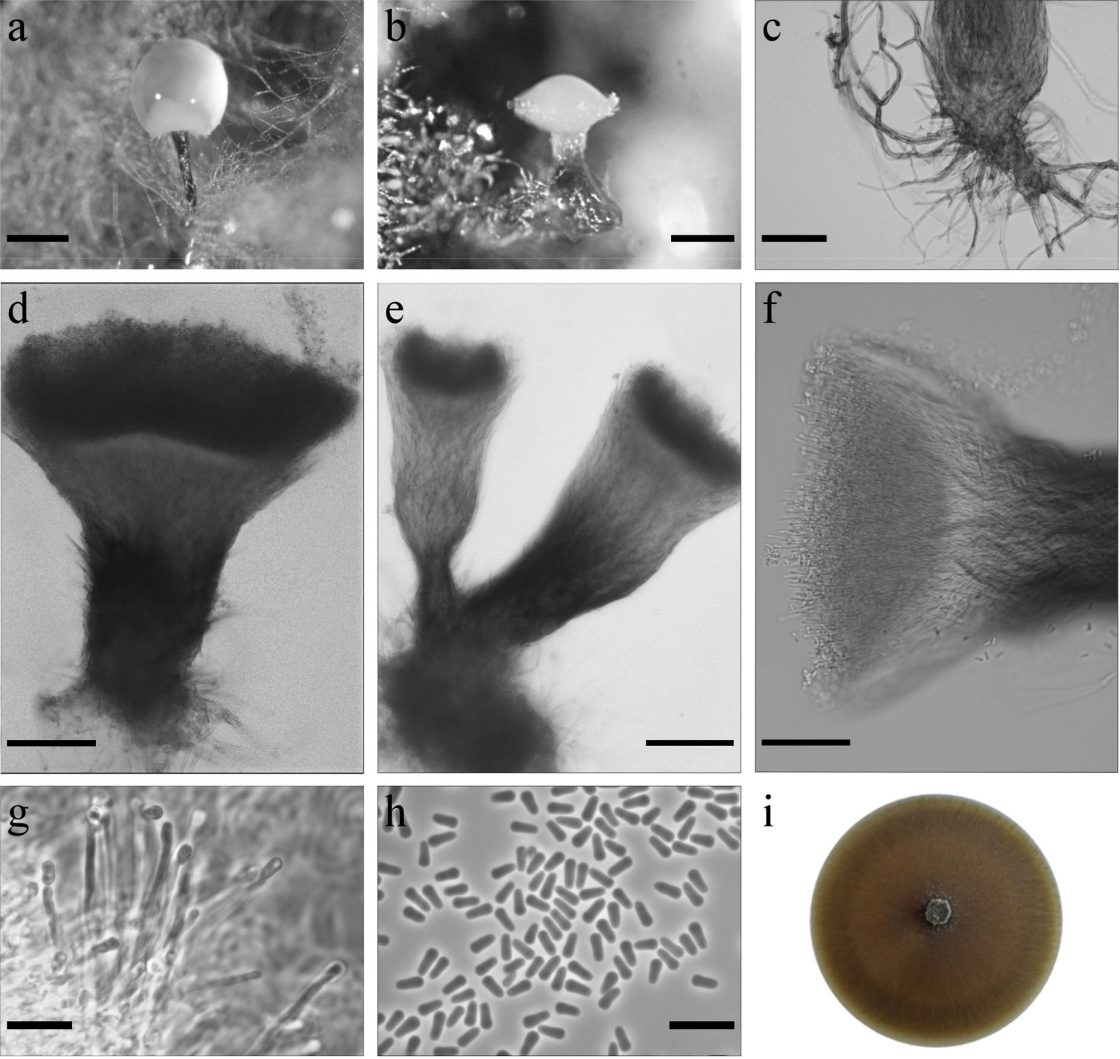
Morphological characters of *Ophiostoma pityokteinis* sp. nov. (CBS 144879, Taxon 2). **a**, **b** Synnematous asexual state on wood tissue; **c** rhizoid-like hyphae of synnemata anamorph; **d**–**f** conidiophores; **g** conidiogeneous cells; **h** conidia; (i) fourteen-day-old culture on MEA. Scale bars: **a** = 250 μm, **b** = 250 μm, **c** = 50 μm, **d** = 100 μm, **e** = 100 μm, **f** = 50 μm, **g** = 10 μm, **h** = 10 μm

*Ophiostoma pityokteinis* R. Jankowiak & P. Bilański, sp. nov. (Fig. 6). MycoBank: MB830196

*Etymology* The epithet pityokteinis reflects the genus name of the bark beetle vector for this fungus, *Pityokteines*.

*Sexual state* not observed. *Asexual states*: pesotum-like. *Conidiophores* macronematous, synnematous, abundant in culture and sterilized fir twigs (187–)265–511(–698) μm long including conidiogenous apparatus, synnemata occurring singly or in groups, stipe well or poorly developed, light or dark pigmented, mostly expanding towards the apex, cup-or club shaped (101–)133–319(–481) μm long (26–)51–122(–169) μm wide at base, and (29–)50–167(–302) μm wide at apex (Fig. 6a–f). *Conidiogenous cells* discrete, terminal, hyaline, cylindrical (11.1–)15–30.7(–40.6) × (0.6–)0.9–1.3(–1.5) μm (Fig. 6 g). *Conidia* hyaline, one-celled, obovoid (3.2–)3.5–4.1(–4.5) × (0.9–)1.2–1.6(–1.8) μm aggregating into a cream-white mucilaginous spore drop (Fig. 6h). Synnemata anamorph attached to substrate by brown rhizoid-like hyphae (Fig. 6c).

*Culture characteristics* Optimal growth temperature on MEA is 25 °C with radial growth rate of 3.9 (± 0.1) mm/d, no growth occurred at 35 °C. Colonies on MEA hyaline at first, later becoming coffee to dark brown in colour, floccose, with abundant grey aerial mycelium, margin smooth (Fig. 6i). Reverse dark brown. Hyphae light to dark brown in colour (Kornerup and Wanscher 1978), smooth, often fused, submerged in the medium and aerial mycelium abundant, not constricted at the septa, 0.8–5.4 (mean 1.8 ± 1) µm diam.

- Habitat: coniferous forest dominated by *Abies alba*
- Host tree: *Abies alba*
- Insect vectors: *Pityokteines curvidens*, *Pityokteines vorontzowi, Cryphalus piceae* (Jankowiak et al. 2017a)
- Distribution: Poland

*Type material* POLAND, Nawojowa, in coniferous forest dominated by *A. alba*, from *Pityokteines curvidens* beetles infesting *A. alba*, collector *P. Majka*, 17 June 2013. Holotype dried specimen TUR 207546 (http://mus.utu.fi/TFU.207546), ex-holotype living culture CBS 144879 = CMW 52056.

*Notes* This species forms a lineage within *Ophiostoma s. stricto* and can be distinguished from all its members by the ITS (Fig. 2) and protein coding sequences (Figs. S1-S3).

Morphologically, *O. pityokteinis* is most similar to members of the *O. piceae* species complex. In contrast to the species in the *O. piceae* species complex, which produce column-like synnemata, *O. pityokteinis* produces cup- or club-like synnemata. In addition, this species has no sporothrix-like asexual state, which is a characteristic for many species of the *O. piceae* species complex.

*Ophiostoma pityokteinis* has very specific host and vector ranges. It was found abundantly on *A. alba* in associations with fir-infesting bark beetles, especially *Pityokteines* species (Jankowiak et al. 2017a).

### Taxon 3

**Fig. 7:**
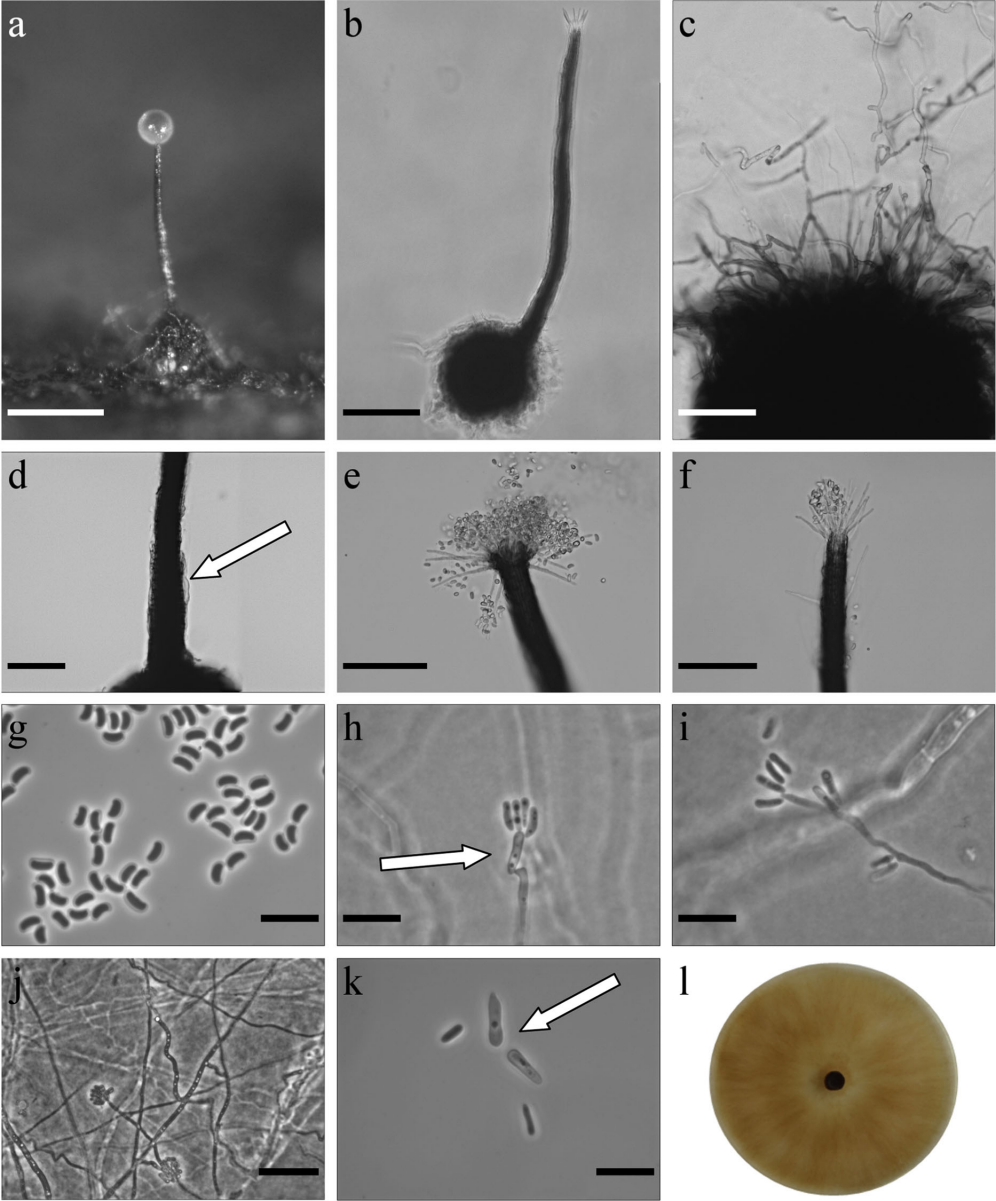
Morphological characters of *Ophiostoma taphrorychi* sp. nov. (CBS 144891, Taxon 3). **a** Ascoma on wood tissue; **b** ascoma; **c** ascomatal base; **d** base of neck, arrow shows paler bumps; **e**, **f** top of neck with ostiolar hyphae **g** ascospores; **h**–**j** conidiophore of hyalorhinocladiella-like asexual state, arrow indicates primary conidium; **k** primary (arrow) and secondary conidia; **l** fourteen-day-old culture on MEA. Scale bars: **a** = 250 μm, **b** = 100 μm, **c** = 50 μm, **d** = 50 μm, **e** = 50 μm, **f** = 50 μm, **g** = 10 μm, **h** = 10 μm, **i** = 10 μm, **j** = 25 μm, **k** = 10 μm

*Ophiostoma taphrorychi* B. Strzałka & R. Jankowiak, sp. nov. (Figure 7) MycoBank: MB830197

*Etymology* The epithet taphrorychi reflects the genus name of the bark beetle vector of this fungus, *T. bicolor*.

*Sexual state* present *Ascomata* abundantly produced on media and sterilized beech twigs, bases black, globose, verrucose (62–)88–130(–169) μm diam., ornamented with brown hyphal hairs of variable length, 5 to 77 μm long and 2.1–3.3 μm wide at the base (Fig. 7a–c). *Necks* black, straight or slightly curved, sometimes with paler bumps, often extended at the base (347–)444–559(–632) μm long with annuli absent or occasionally 1–2 present (Fig. 7d). Diameter of the necks (10.8–)11.8–14.5(–15.4) μm at the apex and (24.6–)29–41.5(49.3) μm at the base. *Ostiolar hyphae* present, pigmented, aseptate, straight, tapering towards the apex (8–)8–12(–15) in number (18.5–)27.7–36.6(–40) μm long (1.2–)1.4–1.7(–1.8) μm at the apex and (2.1–)2.4–3.2–(3.6) μm at the base, sometimes formed below the apex (Fig. 7e, f). *Asci* not observed. *Ascospores* one-celled, allantoid in side view (2.8–)3–3.6(–4) × (0.8–)1–1.3(–1.5) μm, elliptical in front view (2.8–)3.1–3.7(–4) × (1–)1.1–1.5(–1.7) μm and circular in end view to 1.8 (mean 1.5 μm), with residual sheath up to 1 μm thick, accumulated in white-coloured mass at the tip of the neck (Fig. 7 g).

*Asexual state*: hyalorhinocladiella-like. *Conidiophores* mononematous, micronematous, hyaline (9–)11–19(–26) × 1–1.5 µm (Fig. 7h–j). *Conidia* hyaline, smooth, oblong elliptical, obovoid to bacilliform, sometimes allantoid (3.3–)4.2–5.4(–6.6) × (0.8–)0.9–1.3(–1.7) –1.5 µm. Sometimes *conidia* were formed from primary conidia. *Primary conidia* non-septate, hyaline, clavate (6.7–)7.9–11.1(–13.6) × (1.3–)1.6–2.3(–2.8) μm, sometimes producing denticles and giving rise to secondary conidia (Fig. 7k).

*Culture characteristics* Colonies with optimal growth at 20 °C on 2% MEA with a radial growth rate at 4.2 (± 0.1) mm/d; growth at 15 °C was better when compared to growth at 25 °C, and no growth occurred at 30 and 35 °C. Colonies brown, margins smooth (Fig. 7l). Reverse dark brown. Hyphae olive yellow in colour (Kornerup and Wanscher 1978), smooth, submerged in the medium and aerial light greyish mycelium sparse, not constricted at the septa, 0.9–4.9 (mean 2.3 ± 1.1) µm diam.

- Habitat: hardwood forest dominated by *Fagus sylvatica*
- Host tree: *Fagus sylvatica*
- Insect vectors: *Taphrorychus bicolor*
- Distribution: Poland

*Type material* POLAND, Rozpucie, in hardwood forest dominated by *F. sylvatica*, from *T. bicolor* beetles infesting *F. sylvatica*, collector *P. Bilański*, 2 June 2016. Holotype dried specimen TUR 207555 (http://mus.utu.fi/TFU.207555), ex-holotype living culture CBS 144891 = CMW 52045.

*Notes* This species is most closely related to *O. distortum* (Davidson, 1971). However, the DNA sequences of ITS, βT, CAL and TEF1-α (Figs. 2, S1–S3) clearly suggested that *O. taphrorychi* is distinct from *O. distortum*.

*Ophiostoma taphrorychi* morphologically resembles *O. torulosum*, which was described from *T. domesticum* on *F. sylvatica* in Germany (Butin and Zimmermann 1972). However, it can be distinguished from *O. torulosum* by smaller ascospores, and the presence of a different asexual state. *Ophiostoma torulosum* produces sporothrix-like asexual state, while the new species has a hyalorhinocladiella-like morph producing larger primary and in some instances secondary conidia. In addition, *O. taphrorychi* was isolated only from *T. bicolor* (Jankowiak et al. 2019), suggesting a specific association with this bark beetle species.

### Taxon 4

**Fig. 8:**
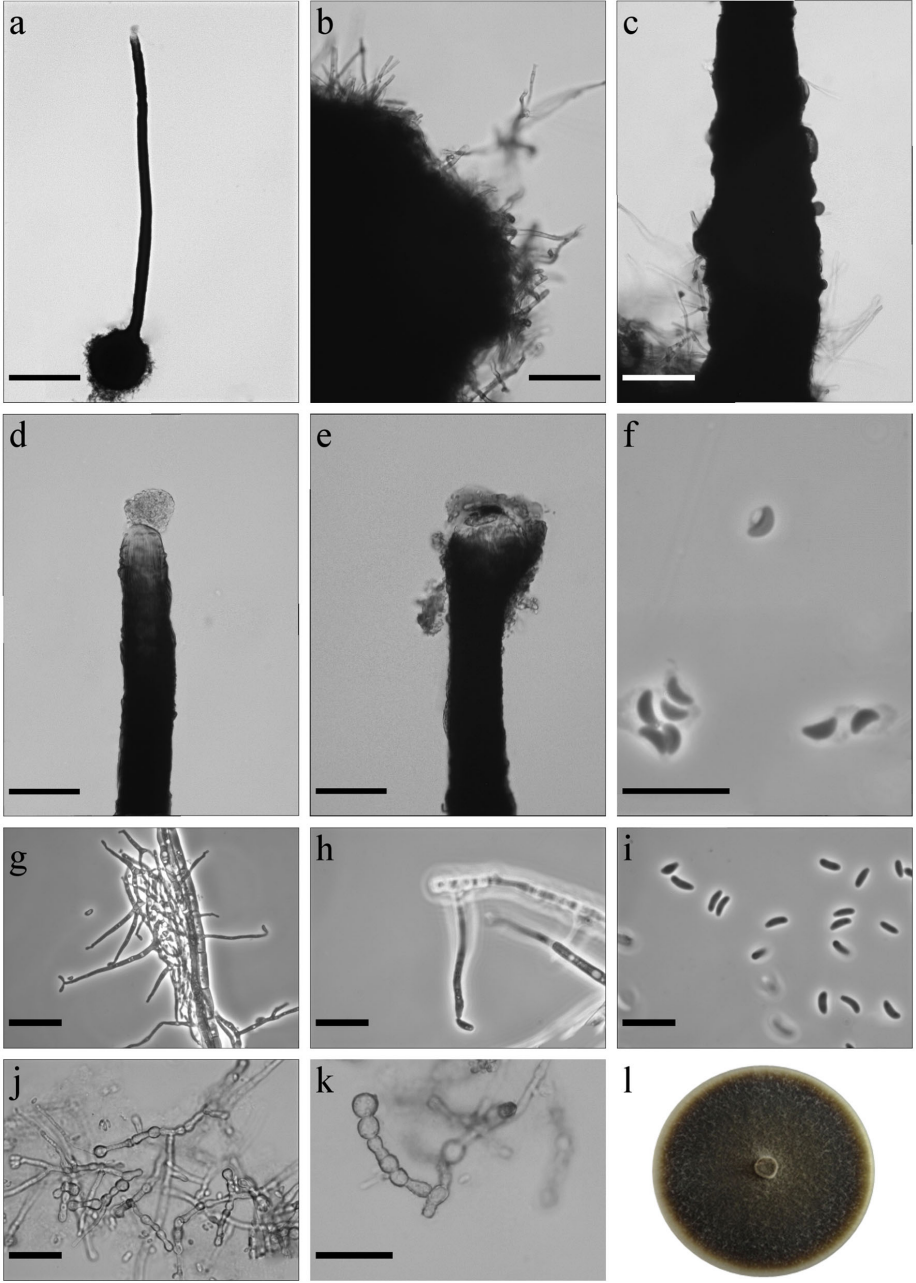
Morphological characters of *Ophiostoma solheimii* sp. nov. (CBS 144881, Taxon 4). **a** Ascoma; **b** ascomatal base; **c** base of neck with bumps; **d** top of neck without ostiolar hyphae; **e** cup-shaped opening at the apex of neck; **f** ascospores; **g**, **h** conidiophore of hyalorhinocladiella-like asexual state; **i** conidia; **j**, **k** chlamydospores-like cells; **l** fourteen-day-old culture on MEA. Scale bars: **a** = 250 μm, **b** = 50 μm, **c** = 50 μm, **d** = 50 μm, **e** = 50 μm, **f** = 10 μm, **g** = 25 μm, **h** = 10 μm, **i** = 10 μm, **j** = 25 μm, **k** = 25 μm

*Ophiostoma solheimii* B. Strzałka & R. Jankowiak sp. nov. (Figure 8). MycoBank: MB830198

*Etymology* Named in honour of Prof. Halvor Solheim, in recognition of the leading role he has played in the study of diversity, ecology and taxonomy of the ophiostomatoid fungi, as well as for his leading role in developing the field of forest pathology in Europe.

*Sexual state* present *Ascomata* abundantly produced on media and on sterilized *Quercus* twigs, bases dark brown to black, globose to ovoid (299–)329–457(–548) μm diam., ornamented with brown hyphal hairs of variable length (13.5–)20.7–65.9(–108.6) μm long, 1.2–2.6 μm wide at the base (Fig. 8a, b). *Necks* black, straight or slightly curved, cylindrical, sometimes with bumps (1192–)1627–2218(–2569) μm long (Fig. 8c). Diameter of the necks (46.5–)51.1–60.9(–67.1) μm at the base and (22.2–)25.1–32.6(39) μm at the apex. *Ostiolar hyphae* not present; sometimes neck forms cup-shaped to funnel-like opening at the apex (Fig. 8d, e). Asci not observed. *Ascospores* one-celled, orange-section shaped in side view (2.5–)2.6–3.1(–3.5) × (0.8–)1.1–1.4(–1.6) μm, ellipsoidal in face view (2.3–)2.7–3.2(–3.8) × (0.8–)1.1–1.6(–1.9) μm, and circular in end view (mean 1.4 μm); sometimes with visible sheath situated towards the inside of the ascospore, to 1 μm thick, accumulated in light brown-coloured mass at the tip of the neck (Fig. 8f).

*Asexual states* hyalorhinocladiella-like. *Conidiophores* mononematous, hyaline, terminal (4.7–)11.7–45.1(–103.3) × (0.6–)0.9–1.8(–2.5) μm (Fig. 8g, h). *Conidia* hyaline, smooth, one-celled, allantoid in side view (2.9–)3.7–5.1(–6.3) × (0.8–)1.1–1.7(–2.2) μm, ellipsoidal in face view (3.1–)3.9–4.8(–5.3) × (0.9–)1.2–1.8(–2.1) μm, and circular in end view (mean 1.5 μm) (Fig. 8i).

*Culture characteristics* Colonies with optimal growth at 25 °C on 2% MEA with radial growth rate 4.0 (± 0.1) mm/d, no growth occurred at 5 °C. Colonies olive brown, margin smooth (Fig. 8l). Hyphae olive yellow in colour (Kornerup and Wanscher 1978), smooth, submerged in the medium and aerial mycelium abundant, not constricted at the septa, 1.1–5.5 (mean 2.5 ± 1) µm diam. Chlamydospore-like cells terminal or intercalary present, in short chains, 3.1–9.9 (mean 5.9 ± 1.6) μm in diameter (Fig. 8j, k). Ascomata and asexual morph co-occur in culture.

- Habitat: hardwood forest dominated by *Quercus robur*
- Host tree: *Quercus robur*
- Insect vectors: *Anisandrus dispar*
- Distribution: Poland

*Type material* POLAND, Resko, in hardwood forest dominated by *Q. robur*, from *A. dispar* beetles infesting *Q. robur*, collector *P. Wieczorek*, 7 October 2016. Holotype dried specimen TUR 207548 (http://mus.utu.fi/TFU.207548), ex-holotype living culture CBS 144881 = CMW 52050.

*Notes* This species is most closely related to *O. grandicarpum* (Kowalski and Butin 1989). However, the DNA sequences of ITS, βT, CAL and TEF1-α (Figs. 2, S4) clearly suggested that *O. solheimii* is distinct from *O. grandicarpum*. Morphologically, *O. grandicarpum* can be distinguished from this new species by distinctly larger perithecia and ascospores. In addition, *O. grandicarpum* has white colonies, while *O. solheimii* has olive brown colonies. *Ophiostoma solheimii* has been infrequently isolated from *Q. robur* L. in association with *A. dispar* (Jankowiak et al. 2019).

## Discussion

In the present study, multigene phylogenies and morphological comparisons revealed four new species of *Ophiostoma s. lato* associated with five species of conifer- and hardwood-infesting bark beetles from the Czech Republic and Poland. These species were described herein as: *Ophiostoma pityokteinis* sp. nov., *Ophiostoma rufum* sp. nov., *Ophiostoma solheimii* sp. nov., and *Ophiostoma taphrorychi* sp. nov.

*O. rufum* sp. nov. was assigned to the *O. piceae* species complex, as defined by Harrington et al. (2001) based on ITS sequence analysis of ten hardwood and conifer-inhabiting synnematous species. The monophyly of this group in Harrington’s studies (2001) was not statistically supported. However, in subsequent studies, ‘hardwood’ species formed a separate lineage with substantial support, a lineage that was subsequently referred to as *O. quercus* species complex (Kamgan Nkuekam et al. 2011) or the *O. ulmi* species complex (De Beer and Wingfield 2013). The conifer-inhabiting species previously included in the *O. piceae* species complex (Harrington et al. 2001; Linnakoski et al. 2010) did not form a monophyletic lineage in recent reports based on ITS and LSU analyses (De Beer and Wingfield 2013; Yin et al. 2016). The monophyly of the *O. piceae* species complex was also not well supported in the present study. Nevertheless, based on individual protein-coding genes, as well as the phylogenetic analysis of the concatenated dataset these species formed a monophyletic lineage with substantial support. Yin et al. (2016) recommended the designation of a newly defined *O. piceae* species complex. They also noted that members of the *O. piceae* species complex have similar morphological characteristics such as unsheathed, allantoid ascospores, and pesotum-like synnemata and sporothrix-like asexual morphs. The monophyly of the *O. piceae species* complex based on three protein-coding gene regions, including βT, CAL and TEF1-α sequence data was confirmed in the present study and *O. rufum* fits well into this species complex as a conifer-inhabiting species forming pesotum- and sporothrix-like asexual morphs.

*O. rufum* is highly similar to *O. breviusculum*, which was originally described from *Ips subelongatus* and *Dryocoetes baikalicus* infesting Japanese larch (*Larix kaempferi*) in Japan (Chung et al. 2006). The colony morphology on MEA is the main morphological difference between *O. rufum* and *O. breviusculum*. In addition, *O. rufum* produces shorter synnemata compared to *O. breviusculum*, and has unique crystalline structures in the upper part of the stipe. *Ophiostoma breviusculum* is considered heterothallic (Chung et al. 2006). Although we were not able to observe the sexual state, one would infer that *O. rufum* is also heterothallic. *Ophiostoma rufum* and *O. breviusculum* are also quite similar based on their host range and beetle vectors. Both species appear to be associated with *Larix* species (*O. rufum* with *L. decidua*, while *O. breviusculum* with *L. kaempferi*), and *Ips* species (*O. rufum* with *I. cembrae,* while *O. breviusculum* with *I. subelongatus*). However, the DNA sequences of βT and TEF1-α obtained in this study clearly suggested that *O. rufum* is distinct from *O. breviusculum*.

The present study shows that *O. pityokteinis* sp. nov. has a unique ITS sequence. It forms a lineage within *Ophiostoma s. stricto*, grouping close to the *O. piceae* species complex and the *O. distortum* lineage. This new species is characterized by cup- or club-like synnemata, and the lack of a sporothrix-like anamorph. Sexual states were not observed for this species in crosses done between different isolates, suggesting that this species could be heterothallic. *O. pityokteinis* also has unique ecological characteristics; this fungus appears to be commonly associated with bark beetles infesting *A. alba*. In our previous study (Jankowiak et al. 2017a), *O. pityokteinis* was often found in association with *Pityokteines* species infesting *A. alba* in Poland (named as *O.* sp. 2), indicating that it might be a consistent fungal associate of this bark beetle species.

*O. taphrorychi* sp. nov. together with *O. distortum* are morphologically different from the other species in the *O. piceae* species complex and *O. pityokteinis*, and based on molecular data grouped in a distinct lineage within *Ophiostoma s. stricto*. In contrast to the members of the *O. piceae* species complex, *O. taphrorychi* and *O. distortum* do not produce pesotum-like synnemata, rather only sporothrix- or hyalorhinocladiella-like asexual morphs. Examination of additional isolates is needed to resolve the status of this apparently new clade or species complex.

*O. taphrorychi* is morphologically similar to *O. distortum* sensu Yin et al. (2016). A recent study has revealed that *O. arduennense* and *O. torulosum* are synonyms of *O. distortum* (Yin et al. 2016). However, there are some morphological and ecological differences, mainly between the asexual states. No asexual morph is known for *O. arduennense* (Carlier et al. 2006), while *O. torulosum* and *O. distortum* produce sporothrix-like morphs that differ in their conidial size and shape (Butin and Zimmermann 1972; Davidson 1971). Ecologically, *O. arduennense* and *O. torulosum* have been found in association with ambrosia beetles infesting *F. sylvatica* (Butin and Zimmermann 1972; Carlier et al. 2006), while *O. distortum* was described from *Pityokteines sparsus* infesting various conifer trees and unknown ambrosia beetle species (Davidson 1971). Morphologically, *O. taphrorychi* should be compared to *O. torulosum*, and to a lesser degree with *O. arduennense*. *Ophiostoma torulosum* can be distinguished from *O. taphrorychi* by forming ascomata in concentric rings, larger ascospores, and shorter ostiolar hyphae. In addition, *O. torulosum* produces a sporothrix-like asexual state; while *O. taphrorychi* has a hyalorhinocladiella-like morph producing larger primary or secondary conidia. *Ophiostoma taphrorychi* (previously referred as *Ophiostoma* sp. 8) appears to be closely associated with *T. bicolor* on *F. sylvatica* (Jankowiak et al. 2019). This species resides together with *O. distortum* in a discrete, well-supported lineage in *Ophiostoma s. stricto*.

In the present study, *O. solheimii* sp. nov. together with *O. grandicarpum* and *O. microsporum* resided in a well-supported phylogenetic group referred to as lineage A by De Beer et al. (2016) supporting the view that this lineage probably represents a distinct genus in the Ophiostomatales (De Beer and Wingfield 2013; De Beer et al. 2016). Species in this lineage have small, orange-section shaped ascospores without noticeable sheath, and ascomata with very long necks without ostiolar hyphae (sometimes necks form cup-shaped to funnel-like openings at the apex). In addition, these fungi have hyalorhinocladiella-like asexual morphs (Davidson 1942; Kowalski and Butin 1989).

*O. solheimii* is morphologically most similar to *O. grandicarpum* (Kowalski and Butin 1989). These two fungi have homothallic mating systems and perithecia with long necks, however *O. grandicarpum* forms substantially larger ascomatal bases (up to 950 μm in diam) and longer perithecial necks (up to 10 000 μm) compared to *O. solheimii*. In addition, *O. solheimii* has smaller ascospores and cup-shaped to funnel-like openings at the apex of the perithecial necks. Both species produce hyalorhinocladiella-like asexual morphs. However, *O. solheimii* has smaller conidia than *O. grandicarpum*. In addition, *O. solheimii* can also be distinguished from *O. grandicarpum* by colony characteristics. The new species has olive brown colonies, while *O. grandicarpum* forms white to cream coloured colonies. Both species inhabit similar ecological niches. *O. grandicarpum* is known to occur mainly on *Q. robur* in Poland, the Czech Republic, Germany and Russia (Kehr and Wulf 1993; Kowalski and Butin 1989; Kowalski 1991; Novotný and Šrůtka 2004; Selochnik et al. 2015). The fungus was only rarely isolated from *A. dispar* and *Scolytus intricatus* on *Q. robur* in our previous study (Jankowiak et al. 2019). *O. solheimii* (previously referred as *Ophiostoma* sp. 9) so far has been found only in association with *A. dispar* on *Q. robur* (Jankowiak et al. 2019).

Recent surveys of conifer and hardwood-infesting bark beetles conducted in Czech Republic and Poland revealed many potentially new fungal species and new beetle-fungus associations (Jankowiak et al. 2017a, 2019). These findings clearly show that the Ophiostomatales associated with bark and wood-dwelling beetles in Central Europe are very diverse and still poorly understood. In this study, we described four new taxa, which support the view that the diversity of these fungi is likely much higher than currently appreciated. Due to the economic impact of the Ophiostomatales it is important to formally describe species of Ophiostomatales that still remain undescribed. This will allow for a better understanding of the taxonomic status and diversity of these economically and ecologically important fungi.

## Electronic supplementary material

Below is the link to the electronic supplementary material.

**Fig. S1** ML based tree topology for species in the *Ophiostoma piceae* species complex generated from the DNA sequences of βT gene regions. Bootstrap values ≥ 75% for ML and Maximum Parsimony (MP) analyses are indicated at the nodes as follows: ML/MP. Bold branches indicate posterior probabilities values ≥ 0.95 as obtained from Bayesian Inference (BI) analyses. The symbol * denotes nodes with bootstrap values < 75%. The tree is drawn to scale (see bar) with branch length measured in the number of substitutions per site. Taxon 1– *Ophiostoma rufum* sp. nov., Taxon 2 – *Ophiostoma pityokteinis* sp. nov., Taxon 3 – *Ophiostoma taphrorychi* sp. nov. **Fig. S2** ML based tree topology for species in the *Ophiostoma piceae* species complex generated from the DNA sequences for TEF1-α gene regions. The Bootstrap values ≥ 75% for ML and Maximum Parsimony (MP) analyses are shown at the nodes as follows: ML/MP. Bold branches indicate posterior probabilities values ≥ 0.95 as obtained from Bayesian Inference (BI) analyses. The symbol * indicates bootstrap values < 75%. The tree is drawn to scale (see bar) with branch length measured in the number of substitutions per site. Taxon 1– *Ophiostoma rufum* sp. nov., Taxon 2 – *Ophiostoma pityokteinis* sp. nov., Taxon 3 – *Ophiostoma taphrorychi* sp. nov. **Fig. S3** ML based tree topology for species in the *Ophiostoma piceae* species complex generated from the DNA sequences of the CAL gene region. Bootstrap values ≥ 75% obtained for ML and Maximum Parsimony (MP) analyses are indicated at nodes as follows: ML/MP. Bold branches indicate posterior probabilities values ≥ 0.95 were obtained from Bayesian Inference (BI) analyses. The symbol *labels nodes with bootstrap values < 75%. The tree is drawn to scale (see bar) with branch length measured in the number of substitutions per site. Taxon 1– *Ophiostoma rufum* sp. nov., Taxon 2 – *Ophiostoma pityokteinis* sp. nov., Taxon 3 – *Ophiostoma taphrorychi* sp. nov. **Fig. S4** ML based tree topology for species in the *Ophiostoma* lineage A (De Beer 2016) generated from the DNA sequences for the βT (a) and the TEF1-α (b) gene regions. The Bootstrap values ≥ 75% as obtained for ML and Maximum Parsimony (MP) analyses are presented at nodes as follows: ML/MP. Bold branches indicate posterior probabilities values ≥ 0.95 as obtained from Bayesian Inference (BI) analyses. The symbol * defines bootstrap values < 75%. The tree is drawn to scale (see bar) with branch length measured in the number of substitutions per site. Taxon 4 – *Ophiostoma solheimii* sp. nov. (DOCX 148 kb)
