# Supplementary material for: Four new Ophiostoma species associated with conifer- and hardwood-infesting bark and ambrosia beetles from the Czech Republic and Poland
Source: Antonie Van Leeuwenhoek. 2019 May 28;112(10):1501–21. doi: 10.1007/s10482-019-01277-5 (PMC6748885; doi:10.1007/s10482-019-01277-5)
Supplement: Supplementary file 1 — Fig. S1 ML based tree topology for species in the Ophiostoma piceae species complex generated from the DNA sequences of βT gene regions. Bootstrap values ≥ 75% for ML and Maximum Parsimony (MP) analyses are indicated at the nodes as follows: ML/MP. Bold branches indicate posterior probabilities values ≥ 0.95 as obtained from Bayesian Inference (BI) analyses. The symbol * denotes nodes with bootstrap values < 75%. The tree is drawn to scale (see bar) with branch length measured in the number of substitutions per site. Taxon 1– Ophiostoma rufum sp. nov., Taxon 2 – Ophiostoma pityokteinis sp. nov., Taxon 3 – Ophiostoma taphrorychi sp. nov. Fig. S2 ML based tree topology for species in the Ophiostoma piceae species complex generated from the DNA sequences for TEF1-α gene regions. The Bootstrap values ≥ 75% for ML and Maximum Parsimony (MP) analyses are shown at the nodes as follows: ML/MP. Bold branches indicate posterior probabilities values ≥ 0.95 as obtained from Bayesian Inference (BI) analyses. The symbol * indicates bootstrap values < 75%. The tree is drawn to scale (see bar) with branch length measured in the number of substitutions per site. Taxon 1– Ophiostoma rufum sp. nov., Taxon 2 – Ophiostoma pityokteinis sp. nov., Taxon 3 – Ophiostoma taphrorychi sp. nov. Fig. S3 ML based tree topology for species in the Ophiostoma piceae species complex generated from the DNA sequences of the CAL gene region. Bootstrap values ≥ 75% obtained for ML and Maximum Parsimony (MP) analyses are indicated at nodes as follows: ML/MP. Bold branches indicate posterior probabilities values ≥ 0.95 were obtained from Bayesian Inference (BI) analyses. The symbol *labels nodes with bootstrap values < 75%. The tree is drawn to scale (see bar) with branch length measured in the number of substitutions per site. Taxon 1– Ophiostoma rufum sp. nov., Taxon 2 – Ophiostoma pityokteinis sp. nov., Taxon 3 – Ophiostoma taphrorychi sp. nov. Fig. S4 ML based tree topology for species in the Ophiostoma [file 10482_2019_1277_MOESM1_ESM.docx]

**Fig. S1**

**Fig. S2**

**Fig. S3**

**Fig. S4**
